# Functional implications of microbial and viral gut metagenome changes in early stage L-DOPA-naïve Parkinson’s disease patients

**DOI:** 10.1186/s13073-017-0428-y

**Published:** 2017-04-28

**Authors:** J. R. Bedarf, F. Hildebrand, L. P. Coelho, S. Sunagawa, M. Bahram, F. Goeser, P. Bork, U. Wüllner

**Affiliations:** 1 Department of Neurology, University of Bonn, Bonn, Germany; 2 German Centre for neurodegenerative disease research (DZNE), Bonn, Germany; 3 Department of Internal Medicine I, University of Bonn, Bonn, Germany; 4 European Molecular Biology Laboratory, EMBL, Heidelberg, Germany; 5 ETH Zurich, Institute of Microbiology, Vladimir-Prelog-1-5/10, 8093 Zurich, Switzerland; 6 Molecular Medicine Partnership Unit (MMPU), University of Heidelberg and European Molecular Biology Laboratory, Heidelberg, Germany; 7 Max Delbrück Centre for Molecular Medicine, 13125 Berlin, Germany; 8 Department of Bioinformatics, University of Würzburg, 97074 Würzburg, Germany; 9 Evolutionary Biology Centre, Uppsala University, Norbyva ¨gen 18D, 75236 Uppsala, Sweden; 10 Institute of Ecology and Earth Sciences, University of Tartu, 40 Lai St., 51005 Tartu, Estonia; 11 German Center for Infection Research (DZIF), Bonn-Cologne, Germany; 12 Sigmund-Freud-Str. 25, 53127 Bonn, Germany; 13 Meyerhofstraße 1, 69117 Heidelberg, Germany

**Keywords:** Microbiome, Bacteria, Archaea, Viruses, Parkinson, Enteric nervous system, Gut-brain axis

## Abstract

**Background:**

Parkinson’s disease (PD) presently is conceptualized as a protein aggregation disease in which pathology involves both the enteric and the central nervous system, possibly spreading from one to another via the vagus nerves. As gastrointestinal dysfunction often precedes or parallels motor symptoms, the enteric system with its vast diversity of microorganisms may be involved in PD pathogenesis. Alterations in the enteric microbial taxonomic level of L-DOPA-naïve PD patients might also serve as a biomarker.

**Methods:**

We performed metagenomic shotgun analyses and compared the fecal microbiomes of 31 early stage, L-DOPA-naïve PD patients to 28 age-matched controls.

**Results:**

We found increased Verrucomicrobiaceae (*Akkermansia muciniphila*) and unclassified Firmicutes, whereas Prevotellaceae (*Prevotella copri*) and Erysipelotrichaceae (*Eubacterium biforme*) were markedly lowered in PD samples. The observed differences could reliably separate PD from control with a ROC-AUC of 0.84. Functional analyses of the metagenomes revealed differences in microbiota metabolism in PD involving the ẞ-glucuronate and tryptophan metabolism. While the abundances of prophages and plasmids did not differ between PD and controls, total virus abundance was decreased in PD participants. Based on our analyses, the intake of either a MAO inhibitor, amantadine, or a dopamine agonist (which in summary relates to 90% of PD patients) had no overall influence on taxa abundance or microbial functions.

**Conclusions:**

Our data revealed differences of colonic microbiota and of microbiota metabolism between PD patients and controls at an unprecedented detail not achievable through 16S sequencing. The findings point to a yet unappreciated aspect of PD, possibly involving the intestinal barrier function and immune function in PD patients. The influence of the parkinsonian medication should be further investigated in the future in larger cohorts.

**Electronic supplementary material:**

The online version of this article (doi:10.1186/s13073-017-0428-y) contains supplementary material, which is available to authorized users.

## Background

Idiopathic Parkinson’s disease (PD) disease is conceptualized as a progressive protein aggregation disease with the formation of neuronal cytoplasmic aggregates of misfolded α-synuclein (α-syn) and other proteins as the neuropathological hallmark (Lewy bodies [LB]) [1]. LB are present not only in the central nervous system (CNS) but also in the enteric nervous system (ENS) of the entire gastrointestinal tract, corresponding to the clinical notion that the gastrointestinal tract is involved in PD [2].

Lewy, in his original thesis work in 1913, already identified the dorsal motor nucleus of the vagus as a hotspot of brain pathology and Braak et al. more recently confirmed the early involvement of the vagus and hypothesized that PD might originate in the gut and that α-syn aggregation might spread via vagal structures into the CNS and higher cortical regions [3]. The importance of the vagus for partly bi-directional interactions of the ENS and the CNS (“gut-brain axis”) [4–6] has become even more evident since the identification of the cholinergic anti-inflammatory pathway [7]. In line with this direct connection of gut and brain neurons, increasing evidence from cell culture and animal experiments seems to support the hypothesis of spreading or seeding of α-syn oligomers [8, 9]. The deposition of α-syn within the colon can help to distinguish PD patients from controls [10–13]. Nevertheless, when assessed with conventional immunohistochemistry, its diagnostic value as a biomarker has not been finally confirmed as α-syn staining in colonic mucosa was likewise found in PD patients and controls [14], which not necessarily refutes the concept of an intestinal origin of PD pathogenesis. Thus, additional yet unidentified factors beyond α-syn must be involved in the presumed PD disease process.

The gastrointestinal microbiome encompasses a vast diversity of bacterial species and may be regarded as an extracorporeal organ system, which interacts with its host in unprecedented ways just being unraveled now [15]. Severely disturbed gut homeostasis is detrimental for the host but the effects of smaller changes or differences in species variation for nutrition [16, 17], behavior [18], and drug metabolism [19] are just beginning to emerge. Recent studies already linked an altered microbiome to PD, but most participants were well advanced and treated with L-DOPA [20–22], which affects colonic motility and may promote intestinal bacterial overgrowth [23].

Using metagenomic shotgun analysis, we found that early, L-DOPA-naïve PD patients carry an altered gut microbiota composition, i.e. specific taxonomic groups, among others related to intestinal barrier and immune functions, were overrepresented or underrepresented. Functional analyses also suggested differences in microbiota ẞ-glucuronate and tryptophan degrading pathways. Moreover, total virus abundance was decreased in PD participants.

## Methods

### Study participants and clinical characteristics

This study was approved by the local ethics committee of the University of Bonn and all participants gave informed consent (internal ethics vote 126/02). Study participants were recruited from the Department of Neurology at the University of Bonn. To reduce any potential gender effects, we included only male participants in the study. Thirty-one male PD patients (diagnosed according to the UK Brain Bank criteria [24]) were compared to 28 male age-matched non-parkinsonian controls.

Disease severity was measured using the Unified PD Disease Rating Scale (UPDRS part III). Gastrointestinal symptoms and presence of constipation were assessed with a modified version of an interview-based Gastrointestinal Symptom Rating Scale (GSRS) (selected items: borborygmus, abdominal distension, increased flatus, decreased passage of stools, increased passage of stools, loose stools, hard stools, urgent need for defecation and feeling of incomplete evacuation, each item was rated 0–3 according to intensity, frequency, duration or social impact depending on the respective item, Additional file 1) [25]. To avoid alterations of gut microbiota related to either gastrointestinal dysfunction of late stage PD or L-DOPA-induced intestinal effects, we included only early stage PD participants (onset of motor symptoms and diagnosis of PD within the past year) who were naïve to L-DOPA therapy. Further exclusion criteria were: (1) chronic and inflammatory gastrointestinal diseases including chronic constipation; (2) the use of laxatives or immunosuppressive agents in the past three months; (3) atypical or secondary parkinsonism; while (4) the use of antibiotics in the past three months in principle was an exclusion criterion; however, we included three PD patients and three controls despite the intake of antibiotics for one to three days in a period of 28–34 days prior to feces sampling as the omission of those cases from the analyses did not change any result (see also Additional file 2).

Controls were matched regarding general demographics (Table 1, general demographics). Gastrointestinal symptoms were comparable in both groups; in particular, no relevant constipation was present in PD participants; dietary and smoking habits did not differ between groups. No detailed dietary plan was requested prior to the feces collection and samples were collected as first bowel movement of the day.

**Table 1:** Clinical characteristics and general demographic parameters of study participants

	PD	Control	*P* value
Demographics			
n	31	28	
Age (years, mean ± SD)	64.8 ± 9.5	65.6 ± 10.4	0.970
Clinical data			
UPDRS III (mean ± SD)	12.6 ± 6.9	0 ± 0	<0.001
GIT symptoms incl. constipation (GSRS, mean ± SD)	3.4 ± 2.9	2.2 ± 2.0	0.172
Total serum bilirubin	0.23 ± 0.03	0.23 ± 0.03	0.593
Nutritional habits			
Diet			
Omnivorous	30 [96.8%]	28 [100%]	
Vegetarian	1 [3.2%]	0 [0%]	
Probiotics	4 [12.9%]	1 [3.6%]	
Medication			
Amantadine	26 [83.9%]	0 [0%]	
Dopamine agonist	11 [35.5%]	0 [0%]	
MAO inhibitor	28 [90.3%]	0 [0%]	
L-DOPA	0 [0%]	0 [0%]	
Statin intake	1 [3.2%]	11 [39.3%]	
Metformin	1 [3.2%]	3 [10.7%]	
Acetylsalicylic acid	2 [6.5%]	7 [25.0%]	
Smoking			
No	10 [32.3%]	9 [32.1%]	
Yes	5 [16.1%]	4 [14.3%]	
Ex-smoker	15 [48.4%]	15 [53.6%]	

*UPDRS* Unified Parkinson’s Disease Rating Scale, *GIT* gastrointestinal, *GSRS* Gastrointestinal Symptoms Rating Scale, *MAO* monoamine oxidase, *L-DOPA* Levodopa

### Analysis of microbiota

Non-invasively obtained stool samples were shotgun sequenced (paired end) using an Illumina Hiseq4000 and further analyzed with the MOCAT2 pipeline [26]. Briefly, taxonomic mapping quality-controlled generated profiles reads (minimum length cutoff 45 bp, minimum quality score cutoff 20, reads matching Illumina adapters, or human genome were removed) to a database of ten universal single-copy marker genes that were extracted from 3496 NCBI reference genomes and 263 human gut metagenomes [27]. For abundance estimates at the species level, we used mOTU (molecular operational taxonomic units) abundances [27] and for genus-level and family-level abundances, individual NCBI taxonomy-annotated marker gene abundances were summed up.

Quality-controlled reads were also mapped against an annotated reference gene catalog for the human gut microbiome. On average, 88% of all reads could be mapped to the reference gene catalogue (Additional file 3). Abundances of individual genes summed up according to their KO annotation in the KEGG (Kyoto Encyclopedia of Genes and Genomes) database. KEGG and GMM (Gut specific Metabolic Modules) pathway [28] abundances were estimated with the same algorithm as in [29], re-implemented in C++, available from github.com/hildebra/Rarefaction. Briefly, we estimated for each metabolic pathway which of several alternatives had the highest coverage given our KO abundance matrix. If the coverage per pathway was higher than 30%, the median abundance of all KOs in this pathway was used to estimate pathway abundance.

To estimate the number of phages and viruses, we choose a reference gene catalog independent approach, as assembly of mobile genetic elements is notoriously error prone and imprecise. MOCAT2 quality filtered reads were mapped against the ACLAME [30] database using Diamond [31] in sensitive mode, after format conversion from fastq to fasta using sdm [32]. Reads mapping with an e-value <1e-7 were considered valid hits against the database and the number of reads mapping to the classes of “Plasmid,” “Prophage,” and “Virus” were counted and normalized by read number.

### Statistical methods

Statistical analysis was conducted in R 3.0.0. For all univariate tests, taxa with less than five reads over all samples were excluded from this analysis to avoid artifacts, similar to the approach in [33]. The matrix was normalized dividing each feature by the respective total sample sum and transformed with log10(x + 1), where x is the normalized feature coverage as calculated in the mOTUs algorithm. For species and mOTU level analysis, we made the filtering options more explicit to exclude spurious correlations: from the abundance matrix features were removed that were absent in more than ten samples, had less than ten accumulated or two mean read coverage. Sample count matrices were rarefied using the R implementation of the rtk toolkit [34]. Significance between groups of samples was tested with a Kruskal–Wallis test, as implemented in R. The ordinations (non-metric multidimensional [NMDS] ordination) and subsequent statistical analysis were calculated using the R-package vegan with Bray-Curtis distance on the rarefied and log-transformed taxa abundance and visualized with custom R scripts. Intergroup differences for the microbiota were calculated using a PERMANOVA test as implemented in vegan [35]. This test compares the intragroup distances to the intergroup distances in a permutation scheme and thus calculates a *P* value. For all PERMANOVA tests we used 4999 randomizations. PERMANOVA post hoc *P* values were corrected for multiple testing using the Benjamini–Hochberg false discovery rate (q-value) [36]. Sample composition plots were visualized with custom R scripts (available from github.com/hildebra/PD_helpers_R).

### Univariate testing

Univariate testing for differential abundances of each taxonomic unit between two or more groups was tested using a Kruskal–Wallis test (*P* value), corrected for multiple testing using the Benjamini–Hochberg false discovery rate (q-value).

The Blocked “independence test” function calls (with the following options: “ytrafo = rank, teststat = scalar” for blocked WRST) were used from the COIN R package [37] to control for potential confounders, such as the intake of a statin.

Post hoc statistical testing for significant differences between all combinations of two groups was conducted only for taxa with a significance of *P* < 0.2. Wilcoxon rank-sum tests were calculated for all possible group combinations and corrected for multiple testing using Benjamini–Hochberg false discovery rate (q-value). GSRS, UPDRS III, and Bilirubin correlations to taxa were tested using a spearman correlation test; *P* values were corrected using Benjamini–Hochberg false discovery rate.

Further, we confirmed these results using ANCOM [38], a statistical test developed for microbial count data, using an R implementation version 1.1-3 and the additional parameters multcorr = 2 and sig = 0.1, that is with multiple testing correction at significance 0.1.

### Classifier

For generating a classifier, genera were filtered, removing any whose mean relative abundance across samples was below 0.1%. Subsequently, relative abundances were normalized by a log-transformation with a pseudo-count equal to one-tenth of the estimated detection limit, estimated as the minimal abundance of any positively detected taxa [39]. Fitting a classifier proceeds in two steps. First, a lasso-penalized logistic regression classifier is used to select the top features (the features with the highest absolute weight are selected, Additional file 4). Second, these features are used in an unpenalized logistic regression classifier. Classifiers were based on the scikit-learn implementation [40]. The classifier was generated using cross-validation in a leave-one-out schedule (feature selection was performed de novo at each iteration to provide unbiased estimates). Relevant taxa were extracted from a model trained on the whole data. P values were computed by the Mann–Whitney test on the prediction scores of the two classes (PD and control). Classifier performance was reported as ROC-AUC, representing the probability that the classifier will correctly label a new sample.

### Structural equation model (SEM) from the same sample distribution

SEMs were used to test causality through examination of both direct and indirect effects of bacteria and medication on PD and vice versa [41]. We also evaluated an alternative model in which medication affects bacteria and bacteria in turn affect the disease. Based on this we used an exploratory approach to approximate a significant model fit. Briefly, non-significant variables and paths were subsequently removed using backward elimination stepwise regression and new paths were added based on modification indices until significant model fit was achieved (Additional file 5). This analysis was performed using the computer program AMOS ver. 7.0 (SPSS, Chicago, IL, USA).

## Results

### Microbiota in PD

Analyzing the two sample sets for differences in their bacterial composition we detected significant differences between PD and control samples (Permanova test, *P* < 0.001, Additional file 6).

**Fig. 1:**
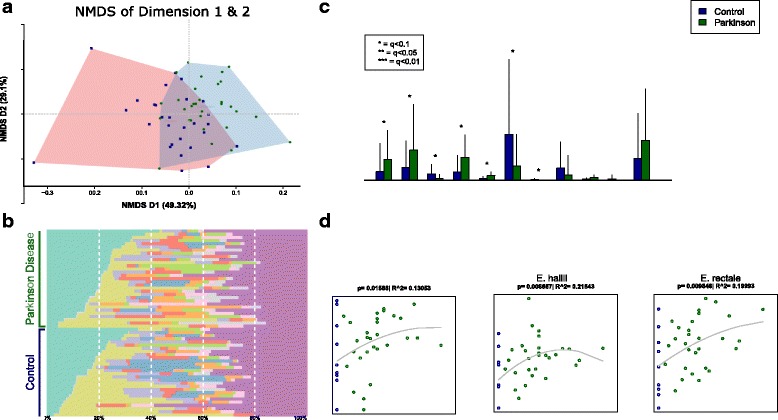
Genus and species level differences in PD participants and controls. **a** NMDS ordination of all samples used in this study, using a Bray–Curtis between-sample distance at genus level. This shows the composition relatedness of samples and that PD samples form a subgroup. Outliers denoted with # took antibiotics in a period of 28–34 days prior to feces sampling. See also Additional file 2 for taxonomic analysis while taking these samples into account. **b** Genus-level sample composition. **c** The most significant species or groups of taxa that could not be further classified. Unclassified Prevotella is not significant after multiple testing, but was implied in PD in several studies (see “Discussion”). **d** Species correlating strongest to PD disease severity (as measured by UPDRS III). Note that after multiple testing correction, these are all q > 0.1

A NMDS ordination separated PD and controls on the first axis of microbiota composition using Bray–Curtis distance, which explained 49.32% of variation, while axes 2 and 3 did not show this separation (Fig. 1a). The composition of PD patient gut microbiota was significantly different from control at all taxonomic levels, while there was little variation within PD and the control group. Richness (i.e. number of taxa present in a sample) between communities was not significantly different, whether samples were pooled or considered as single samples (Additional file 7).

### Key taxonomic differences in the PD gut microbiota

In general, we observed Clostridiales, Bacteroidaceae, and Ruminococcaceae as the most abundant families in both sample sets accounting for 50–52% of the relative family abundance. Univariate tests revealed significant changes in family as well as in genus abundance in PD samples (Additional file 8). Verrucomicrobiaceae (genus *Akkermansia*), unclassified Bacteria (of the classified prokaryotes) and Firmicutes were increased, whereas Prevotellaceae (genus *Prevotella*) and Erysipelotrichaceae (genus *Eubacterium*) were markedly lowered in PD samples (Fig. 1b, Additional file 9a, and b; *P* < 0.05, q < 0.1; with the exception of Prevotella (genus) with a *P* < 0.05 and a q = 0.13, thus being n. s.). Using ANCOM, we similarly found genera Akkermansia, Prevotella, Eubacterium, unknown Bacteria, and unknown Firmicutes to be significantly different between PD and control patients.

Metagenomics provides species resolution. Among the classified bacteria, we thus identified a pattern of significantly increased key species in PD including *Akkermansia muciniphila* and *Alistipes shahii*. On the other hand, *Prevotella copri*, *Eubacterium biforme*, and *Clostridium saccharolyticum* were decreased (Fig. 1b and c).

### Correlation of microbiota with PD clinical scores

No significant taxonomic associations were detected, neither at genus nor at species level, when microbiota abundance was correlated with clinical data (UPDRS III, GSRS, or total serum bilirubin, despite of the presumed decreased ẞ-glucuronidation in PD participants, Additional file 10). This was expected, as we had aimed to recruit a cohort of PD patients with a short duration of (motor) symptoms and subjective impairment; nevertheless, some interesting trends (q > 0.1) were observed regarding the symptom severity of PD (UPDRS III) for three different Eubacteria (*E. eligens*, *E. rectale*, and *E. hallii*, Fig. 1d).

Both parkinsonian and gastrointestinal symptoms severity including constipation were rather low in our sample set of early L-DOPA-naïve patients and, except for core parkinsonian features, did not differ from controls (Table 1). We could not detect any deviations pointing to a possible confounder.

### Functional analysis of the PD microbiome

In order to explore differences in the metabolic potential of gut microbiota between PD and control patients, we further estimated the abundance of metabolic pathways, using our metagenomic reads mapped to functional orthologues from the KEGG and GMM databases (Additional file 11).

**Fig. 2:**
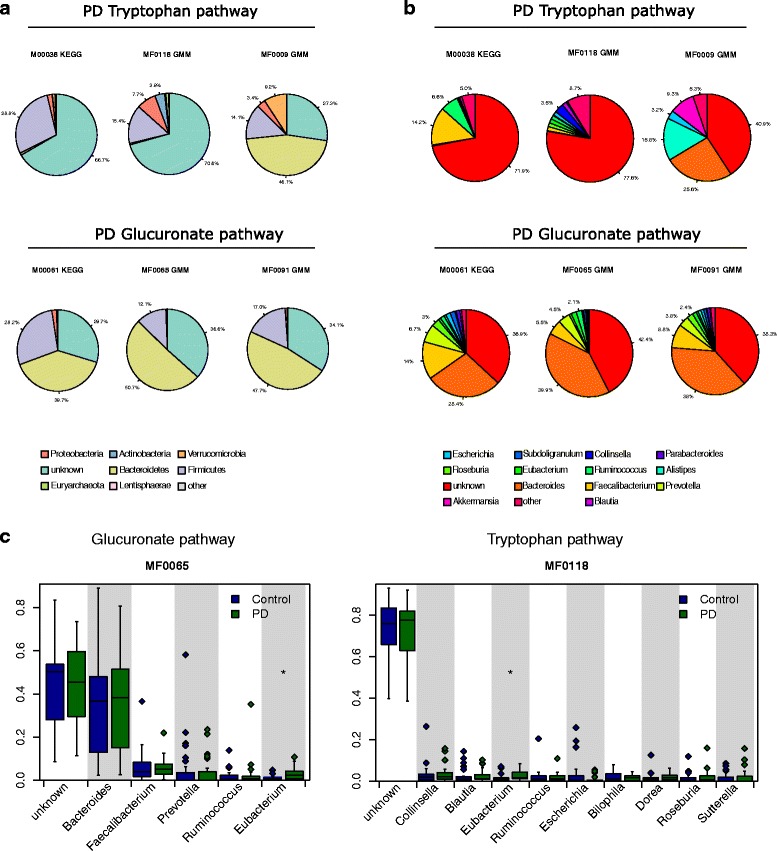
Functional differences in PD patients based on selected metabolic pathways. **a** Phylum and (**b**) genus level composition of six modules that were increased/decreased in PD patients. **c** All genera were contributing to these modules as expected by random chance, with the exception of MF0065 and MF0118, where a higher than expected proportion of reads contributing could be traced to Eubacterium in PD patients

We identified a significantly decreased gene abundance for D-Glucuronate degradation in PD participants compared to controls (System: D-Glucuronate degradation, D-glucuronate → pyruvate and D-glyceraldehyde 3P, Fig. 2a and b, KEGG module number M00061, *P* < 0.05, q < 0.1). GMM Orthology corroborated a decreased abundance of genes in the pathway of beta-D-glucuronide and D-glucuronate degradation in PD participants (Fig. 2a and b, GMM module number MF0091, *P* < 0.05, q < 0.1). This was paralleled by a decrease in genes for 5-dehydro-4-deoxy-D-glucuronate degradation (Fig. 2a and b, GMM module number MF0065, *P* < 0.05, q = 0.11).

Interestingly, we also found two different pathways for tryptophan metabolism, which appeared to be more active in the microbiota of PD participants (tryptophan → kynurenine → 2-aminomuconate, KEGG module number M00038, Fig. 2a and b; tryptophan degradation, MF0009 (GMM module number), Fig. 2a and b, increased formate conversion, GMM module number MF0118, Fig. 2a and b). Although a lack of tryptophan and serotonin is a hallmark of PD, this finding did not reach statistical significance after multiple testing correction (all *p* < 0.05, q > 0.1) and needs further exploration in a larger cohort.

To determine which bacteria are involved in these pathways, we traced the contributing genes and determined their likely taxonomic origin (Fig. 2a and b). Although multiple genera contribute to all modules, only in modules MF0065 and MF0118 could we find evidence of any genera contributing significantly more reads in PD patients than control patients, after multiple testing correction (Fig. 2c). In both cases this was *Eubacterium*.

### Influence of medication on the microbiota

Given the important influence of pharmaceuticals on the gut microbiota [42], we tested the effects of the concomitant PD-specific medication in our cohort (Additional file 12). No significant differences in taxa abundances (families, genera, species) were apparent in the relatively small samples grouped according to the various combinations of anti-PD medication (dopamine agonist + MAO inhibitor + amantadine: *n* = 10, MAO inhibitor + amantadine: *n* = 14, MAO inhibitor: *n* = 3, no therapy: *n* = 3; there was only one patient with dopamine agonist + MAO inhibitor, who was not included). However, patients treated with MAO inhibitors in combination with amantadine (*n* = 14, i.e. 45.2% of the PD participants) displayed a significantly increased richness (Additional file 12d), which did not affect the overall richness of the entire PD cohort.

Among the concomitant drugs the intake of a statin showed an influence on the gut microbiota. We identified five families significantly different between statin-treated and untreated patients (*Burkholderiaceae*, *Propionibacteriaceae*, *Enterococcaceae*, *Actinomycetaceae*, and *Enterobacteriaceae*), none of which contributed to the differences observed between PD participants and controls (Additional files 12a, c and 13).

### Virus abundance is lowered in PD participants

We tested the fraction of the reads that could be mapped to the ACLAME database to estimate the amount of mobile elements in the metagenomes, though this method is limited to known diversity of mobile elements. While the abundances for prophages and plasmids were not different between PD and controls, total virus abundance (reflecting bacterial and archaeal phages) was decreased in PD participants (*P* = 6.7e-5, Additional file 14a). We also found that viruses were increased in participants treated with a statin (*P* = 0.0002), and therefore tested for differences between PD participants and controls, corrected for statin treatment. This again showed a decrease of the virus load in PD participants (*P* = 0.0009, Additional file 14b).

### Gut microbes discriminate PD participants from controls

**Fig. 3:**
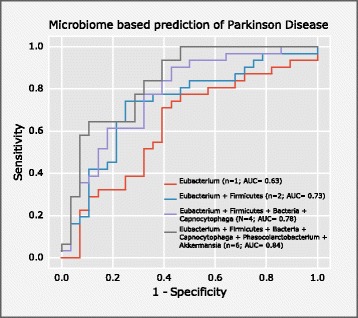
Classification of PD participants based on their microbiome. Here the classifier selected a very similar set of features as were determined by univariate testing (Fig. 1). With only six genera, PD could be separated from control patients with an AUC of 0.84. The exact mOTU composition of each feature is given in Additional file 15

Using a logistic regression classifier to select predictive features, we could discriminate PD from control with a cross-validated AUC of 0.84 using six different taxa (*Eubacterium*, *Capnocytophaga*, *Phascolarctobacterium*, *Akkermansia*, and mOTUs no further classified than to *Firmicutes* as well as *Bacteria level*); this corresponds to a sensitivity of 64.5% and a specificity of 89.2% (*P* value = 4.19 × 10^−6^; Fig. 3, see also Additional files 4 and 15). Eubacterium was frequently selected as most important genus, singularly predicting PD with an AUC of 0.63 (sensitivity = 74.2%, specificity = 57.1%, *P* value = 0.047). The addition of constipation scores as a putative predictive feature showed no added predictive value over the six taxa used, contrary to prior studies [20].

### Structural equation model

**Fig. 4:**
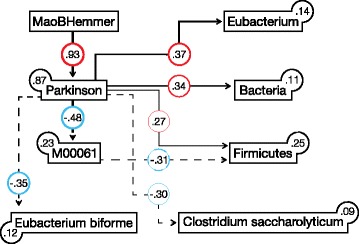
Structural equation modeling (SEM). SEM analysis of PD in relation to key correlating bacterial functions and taxa (MSEA = 0, PCLOSE = 0.79, AIC = 59.385). Values on *paths* and *boxes* are standardized regression and determination coefficients (R2), respectively. *Dashed lines* and *red colors* denote negative relationships. The thickness of lines is proportional to regression coefficients. All relationships are statistically significant (*P* < 0.05, Additional file 5). *AIC* Akaike information criterion, *MSEA* mean square error of approximation, *PCLOSE* probability of close fit

Using a SEM (see “Methods”), we summarized the observed differences in key taxa abundances, metadata, and functional pathways in a holistic statistical model of the PD gut microbiota (Fig. 4). Compared with the LASSO regression classifier mentioned above, this modeling approach enables causality inference through testing both direct and indirect correlations between biotic factors (e.g. bacterial abundance and metadata, e.g. medication), until the model reaches a significant fit through optimization of the correlation network. The best-fit SEM supported our hypothesis that medication used for the studied patients has no direct effect on bacteria, explaining 87% of variation. This model indicated that differences in taxa abundances and also functional changes in ẞ-glucuronidation are basically driven by PD and not vice versa with an Akaike information criterion (AIC) of 59.4 (Additional file 4), while the alternate model (PD is partly driven by biotic factors) had an AIC of 67.5 (Additional file 16). None of the taxa seems to influence the PD microbiota. However, in this model the PD microbiota could be influenced by the intake of a MAO inhibitor (that was only taken by a fraction of participants). Key taxa in this model reflect changes on species levels. Among taxa themselves, the strongest positive correlation was found for *Eubacterium* and unclassified Firmicutes, when correcting for cross interactions.

## Discussion

### Main results

Our results show a significantly altered microbial composition in early disease stage L-DOPA-naïve PD participants. Compared to earlier studies investigating microbiota in PD patients using 16S-based techniques the chosen methods allowed to detect changes at the species level and also a decreased virus load in PD.

We confirmed a decrease of *Prevotella copri* in PD and in addition found decreased *Eubacterium biforme* and *Clostridium saccharolyticum* and increased *Akkermansia muciniphila* as well as *Alistipes shahii*. Based on the taxonomic differences alone, logistic regression after feature selection allowed separation of PD patients in early stages from controls with good accuracy (AUC 0.84). Furthermore, our analyses point to differences in microbiota metabolism, namely in ẞ-glucuronate and tryptophan degrading pathways.

### Taxa abundances

Despite several differences in study design compared to previous work [20, 21], our results strengthen the hypothesis of a PD-specific “microbial footprint.” The observed differences though are not directly comparable, since the previous studies were using 16S amplicon sequencing with its various biases [43], while we used metagenomics with increased precision due to usage of single copy marker genes with high confidence taxonomic assignments [27]. Testing for causality inference of biotic and abiotic factors seem to indicate that these taxonomic changes are a consequence of the disease. However, whether the observed changes are primary changes or rather secondary, resulting from unidentified effects, and whether it is either beneficial or harmful cannot be decided at present.

The increase in *Akkermansia muciniphila*, which appears to be in certain consistency in regards to the findings of Keshavarzian et al., Scheperjans et al., and Unger et al. [20–22], may illustrate this dilemma: it is a common mucin degrader which has been shown to reverse diet induced pathological intestinal changes in high-fat fed mice by restoring the intestinal mucus layer and the underlying epithelium, thus being able to improve gut barrier function [44, 45]. Protective effects of extracellular vesicles derived from *Akkermansia muciniphila* on experimentally induced colitis further support a beneficial influence on intestinal immunity [46]. On the other hand, inflammatory and regulatory properties have been reported for *Akkermansia*, probably mediated due to an increased exposure of immune cells to microbial antigens upon breaking down the mucosal mucin layer [47]. Preliminary evidence also linked *Akkermansia* to multiple sclerosis [48]. Previous studies on colonic biopsies and feces samples from treated and drug-naïve PD participants suggested an altered mucosal barrier function and PD patients exhibited significantly greater intestinal permeability than controls, paralleled by an increased mucosal staining for *E. coli* and α-syn [49]. Pro-inflammatory dysbiosis may even trigger α-syn misfolding or neuronal injury from gut-derived endotoxins [21, 50]. Although we did not identify *E. coli* species associated with PD in our samples, the increase in *Akkermansia* might be associated with a yet unexplored disease related impact on mucosal barrier function.

Extending the results of Scheperjans et al. and Unger et al. [20, 22], which pointed to a relatively lower abundance of Prevotellaceae in advanced PD, *Prevotella copri* was markedly lowered in our samples of early stage PD. On the other side, Keshavarzian et al. [21] did not show this difference for fecal samples, albeit the trend was the same for mucosal-derived PD samples. However, *Prevotella* abundance was also reduced in Japanese multiple sclerosis patients and in autistic children, somewhat questioning the specificity of this finding [51, 52]. The *Prevotella* enterotype is the least prevalent in human individuals [53] and is related to dietary/fiber intake [54, 55]. In particular, *Prevotella* enrichment has been linked to non-Western and/or fiber-rich diets [56, 57]. Fibers are the primary substrate for short chain fatty acids (SCFAs) including butyrate and reductions in the latter can disrupt barrier function and promote inflammation [58]. The fact that, in various autoimmune diseases including type 1/2 diabetes, irritable bowel disease, rheumatoid arthritis, and Behcet’s disease reduced levels of *Prevotella* have been found [42, 59–61], could indicate a decreased SCFA production (i.e. propionate) and in turn favor inflammatory conditions in PD [21].

In contrast to Scheperjans et al. [16], we did not find increased Ruminococcaceae (phylum Firmicutes) to compensate lower levels of *Prevotella* but instead an increase in unclassified Firmicutes. Interestingly, although the early, L-DOPA-naïve PD patients hold a different species pattern not yet affected by drug effects and the chronic constipation typically observed in late-stage PD, we found a certain consistency with the advanced PD patients’ pattern.

Taken together, the observed bacterial pattern in our PD samples might hint towards yet unexplored mechanisms of a disturbed intestinal and immune function in PD pathogenesis. Colonic biopsies from PD patients indeed showed enhanced pro-inflammatory cytokines and glial markers correlating with disease progression [62, 63]. Furthermore, there is evidence of α-syn contributing to neuro-inflammation by potentiating microglial or astroglial activation [64]. In line with this, recent work highlighted the crucial role of microbiota on maturation and activity of microglia [65], which have been considered as one of the earliest contributors of neurodegeneration [66] and further supports the importance of microbial-derived mediators (gut peptides, chemokines, SCFAs) on immune regulation and CNS function [65, 67].

### A possible role for SCFAs in PD

SCFAs are essential energy sources for colonocytes and reduced levels of SCFAs might not only contribute to a decreased colonic motility (i.e. constipation) but also led to an increase in intestinal barrier leakiness [68–70]. Keshavarzian et al. and Unger et al. both suggested a beneficial role for SCFAs as PD-derived feces were shown to contain less SCFA butyrate-producing bacteria, including Blautia, Roseburia, and Coprococcus [21] as well as Faecalibacterium prausnitzii [21, 22], which were previously attributed to exert putative anti-inflammatory effects.

While SCFA administration contra-intuitively promoted motor dysfunction and α-syn-mediated neuro-inflammation in a germ-free transgenic mouse model over-expressing α-syn, oral administration of heat killed bacteria had no effect on motor performance, indicating the putative importance for metabolically active microbiota in disease pathogenesis [71]. Namely, when PD-derived microbiota (of treatment-naïve new onset PD donors) were orally transferred to germ-free mice, several taxa, including Roseburia, Rikenellaceae, and Enterococcus, were markedly altered in the microbial profile of the recipient mice independently of its genotype as when they received microbiota derived from healthy donors.

While inconclusive at the moment, prospective research on SFCA gene expression and metabolomic profiles of microbiota in health and disease will shed further light on this aspect.

### Viral analyses

The gut bacteria harbor a diverse phageome and virome that may contribute to function and structure of the microbiome, but evidence from comparative analyses of the human gut phageome is limited. Recently a comprehensive metagenomic analysis in 64 individuals suggested a core phageome that was shared among more than one-half of all individuals and might also exert beneficial properties as it was reduced in individuals with inflammatory bowel disease [72]. However, based on our analysis, we could not find any differences in the abundance of prophages and plasmids between PD and control samples. In contrast, total virus amount was significantly lowered in PD participants.

Importantly, the assessment of virus and phage load is entirely dependent on the corresponding protein families being present in the ACLAME database; therefore, we only detect those phages or viruses, of which a closely related reference genome is present in the database. Testing for correlations between bacterial family and viral load abundance showed no significant correlations after multiple testing.

As viruses interact with host cells and influence immune response (i.e. prevent inflammatory conditions [73]), there might be various possibilities in which viruses interact in the pathogenesis of PD. Although inconclusive at the moment, exploration of the specific role of viruses in PD is a promising avenue to follow-up with more specific research.

### Functional aspect

Accumulating evidence suggests a direct impact of metabolic alterations in microbiota on human health [74, 75]. We observed a putative reduction in microbiota ẞ-glucuronidase activity in early stage PD participants. Decreased ẞ-glucuronidation in the microbiota could imply a deterioration of resistance to various pathogenic organisms [76]. Also, microbial derived ẞ-glucuronidases affect effective dose availability of administered drugs by reactivation in the gut, which has been shown for irinotecan therapy in colorectal cancer patients [77]. Altered metabolisms of xenobiotics or parkinsonian pharmaceuticals metabolized in the ẞ-glucuronate degrading pathway must be determined experimentally [78].

Our data further revealed a trend towards an increased tryptophan degradation gene copy number in PD. If one assumes that this increased genetic potential translates into an increased tryptophan metabolization, this finding is in line with previous research and is of particular interest as L-tryptophan, the precursor for serotonin, is decreased in PD patients’ brains. L-tryptophan is also metabolized to kynurenines, whereof metabolites have regulatory immune function and were described as either harmful or beneficial in PD [79–81]. Urinary metabolomics profiling demonstrated significant changes of urinary markers including an increased tryptophan metabolism, which was associated with the progression of PD [82]. Interestingly, catabolism of serotonin also includes glucuronidation in the human intestine [83].

The association with these metabolic pathways point to a deeper involvement of *Eubacteria* with PD. Indeed *Eubacteria spp*. were decreased in PD (*Eubacterium biforme*) and other Eubacteria species (*E. hallii*, *E. rectale*, *E. eligens*) showed a trend towards correlation with disease severity (n.s.). Specifically colonizing the mucus layer, particularly *Eubacterium rectale*, might be interconnected with processes directly affecting the mucus layer due to its ability to gain access via flagella [84]. Further, diversity of *Eubacterium rectale* was also reduced in an in vitro dynamic gut model (M-SHIME) of long-term colonization of the mucin layer when microbiota were derived from ulcerative colitis patients [85]. Additionally, *Eubacterium halii* is viewed as a key species impacting the microbial balance due to its ability to produce several SCFAs [86]. In turn, alterations in the abundance of different *Eubaceria* might contribute to the PD pathogenesis via metabolic but also direct mucosal pathways.

Lowered Eubacteria (family Erysipelotrichaceae) in mucosal as well as in fecal PD samples were similarly observed in the study of Keshavarzian et al. [21]; however, a correlation with disease severity was not proven.

### Clinical aspects

Instead, Keshavarzian et al. found PD duration correlating with the greatest number of taxa, whereby the family Lachnospiraceae, which includes several (supposedly anti-inflammatory) butyrate producing bacteria, displayed a significant negative correlation. Scheperjans et al. further showed a significant association of Enterobacteriaceae with the postural instability and gait disorder (PIGD) PD phenotype [20], which was not confirmed in the work of Unger et al. [22].

Namely, based on our analyses, the intake of different anti-parkinsonian drugs had no overall influence on taxa abundance or microbial functions. However, in future subgroup analyses PD patients under the therapy with MAO-inhibitors and amantadine might be favorably influenced by an increased richness if assessed in a lager cohort. In this context, it is worth noting another study, which was published during the revision process of this manuscript and which demonstrates instead independent effects of different PD medications on the microbiome [87].

However, although the intake of a statin showed an influence on the gut microbiota with, in total, five families being different in statin-treated individuals, none of them contributed to the differences observed between PD participants and controls when controlling for statin intake with differential statistical methods. One caveat of testing for confounders in our cohort is that this result might be limited by the sample size being too small to find even small effects, which is an unavoidable inherent aspect of human cohort studies. However, future studies should address this aspect.

## Conclusions

Our data revealed differences of colonic microbiota between PD patients and controls at an unprecedented detail not achievable through 16S sequencing: altered representation of several taxa including *Eubacterium biforme*, which has not been reported previously and might be limited to detection via metagenomics. The functional differences in the gut microbiota included ẞ-glucuronate and tryptophan degrading pathways. The findings point to a yet-unappreciated aspect of PD, possibly involving the intestinal barrier function and immune function in PD patients. We further show the benefits of integrating functional microbiota predictions into microbial-based profiles to discriminate health and disease that is promising as it holds the potential to identify PD patients. Furthermore, it is now evident that exploration of the PD virus populations is a promising avenue to follow up with more specific research.

## Additional files

Additional file 1: Gastrointestinal Symptom Rating Scale (GSRS). Modified version of the GSRS, Gastrointestinal Symptom Rating Scale according to Svedlund et al. 1988, each item was rated from 0 to 3 according to intensity, frequency, duration, or social impact, respectively. (PDF 12 kb)

Additional file 2: PD microbial differences are not confounded by pre-study antibiotics use. Statistical analysis blocked for six patients that used antibiotics 28–34 days prior to sampling. Excluding these samples from the statistical analyses does not change the results: the PD samples are at genus level still significantly different from the control samples. (XLSX 53 kb)

Additional file 3: Statistics of sequencing. Sequencing statistics for all samples of this study, including the number of reads per sample and the number of reads per sample mapped to the gene catalogue. (XLSX 25 kb)

Additional file 4: Feature weights. Features selected by the Lasso classifier, showing the weights of features at different taxonomic levels that the classifier was trained on. Positive numbers resemble positive association with PD. (XLSX 25 kb)

Additional file 5: Structural equation model (SEM). Estimated parameters of the SEM model and the significance of features to PD. (XLSX 9 kb)

Additional file 6: Permanova test. Permanova test for compositional differences between PD and control patients showed significant differences at all taxonomic levels. In contrast, the compositional dispersion as tested with a betadisper test showed significant differences at no levels, with the exception of the species level (*P* = 0.045). (XLSX 8 kb)

Additional file 7: Richness and mOTUs. **a** Richness of single samples (rarefied to 3000 read coverage) was similar between PD and controls, (**b**) also, pooling samples and rarefying to different depths showed a similar pattern (rarefied to 251189), as well as (**c**) measuring the accumulation of new mOTUs when randomly increasing the sampling space. However, evenness and Shannon diversity were positively correlated to UPDRS III. (PDF 64 kb)

Additional file 8: Univariate Testing. Significant taxa differences between PD participants and controls. (XLSX 34 kb)

Additional file 9: Key taxonomic gut microbiota differences between PD participants and controls. **a** The 11 most abundant families and their contribution to the gut microbiota displayed in a pie chart. **b**, **c** The most significantly different genera and families between PD participants and controls (q < 0.1), confirming previous studies. Note that unclassified bacteria were higher in PD patients. (PDF 88 kb)

Additional file 10: Correlation of microbiota with clinical scores. Correlation of GSRS, UPDRS III and bilirubin to taxa with spearman correlation test and Benjamini–Hochberg false discovery rate correction. Species correlating strongest to PD disease severity are shown in Fig. 1d. (XLSX 97 kb)

Additional file 11: Functional analyses. Functional differences between PD and control patients, using KO and COG enzyme annotations, as well as GMM and KEGG modules. (XLSX 904 kb)

Additional file 12: Microbiota differences linked to medication, especially the intake of a statin seemed to have a strong influence on gut microbiota, with (**a**) five bacterial families as well as (**b**) family richness significantly different between drug users and medication free patients. **c** PD medication did not show significant differences in family composition, while (**d**) gut microbiota mOTU richness differed markedly for patients taking MBI + Aman. *DA* dopamine agonist, *MBI* monoamine oxidase inhibitor, *Aman* amantadine. (PDF 319 kb)

Additional file 13: PD microbial differences are not confounded by statin use. Statistical analysis blocked for statin use. This analysis shows virtually the same families being significantly different between PD and control samples, compared to not controlling for statin intake. (XLSX 14 kb)

Additional file 14: Virus analyses. Fecal virus analyses showed differences between PD and control with (**a**) PD samples containing fewer amounts of viruses, with the 10th, 25th, 50th, 75th, and 90th quantiles being 0.001, 0.001, 0.001, 0.003, and 0.003 for Parkinson samples and 0.001, 0.002, 0.003, 0.011, and 0.016 for control samples, respectively. **b** A link to medication with a statin to increase the content of viruses. (PDF 47 kb)

Additional file 15: mOTU phylogeny for predictive features. The most predictive taxonomic features for PD (Fig. 3) were broken down into the mOTUs [27] comprising them. Further, the percentage to which a given feature was made up by each mOTU is indicated. (XLSX 10 kb)

Additional file 16: Alternative SEM model. Alternative SEM model of PD in relation to key biotic and abiotic factors (MSEA = 0.113, PCLOSE = 0.138, AIC = 67.447) in which PD is driven by biotic factors, had a worse AIC fit than our proposed SEM modeling of PD disease associations (Fig. 4). *AIC* Akaike information criterion, *MSEA* mean square error of approximation, *PCLOSE* probability of close fit. (PDF 192 kb)
