# Erratum to: Functional implications of microbial and viral gut metagenome changes in early stage L-DOPA-naïve Parkinson’s disease patients

**DOI:** 10.1186/s13073-017-0451-z

**Published:** 2017-06-29

**Authors:** J. R. Bedarf, F. Hildebrand, L. P. Coelho, S. Sunagawa, M. Bahram, F. Goeser, P. Bork, U. Wüllner

**Affiliations:** 1 Department of Neurology, University of Bonn, Bonn, Germany; 2 German Centre for neurodegenerative disease research (DZNE), Bonn, Germany; 3 Department of Internal Medicine I, University of Bonn, Bonn, Germany; 4 European Molecular Biology Laboratory, EMBL, Heidelberg, Germany; 5 ETH Zurich, Institute of Microbiology, Vladimir-Prelog-1-5/10, 8093 Zurich, Switzerland; 6 Molecular Medicine Partnership Unit (MMPU), University of Heidelberg and European Molecular Biology Laboratory, Heidelberg, Germany; 7 Max Delbrück Centre for Molecular Medicine, 13125 Berlin, Germany; 8 Department of Bioinformatics, University of Würzburg, 97074 Würzburg, Germany; 9 Evolutionary Biology Centre, Uppsala University, Norbyva gen 18D, 75236 Uppsala, Sweden; 10 Institute of Ecology and Earth Sciences, University of Tartu, 40 Lai St., 51005 Tartu, Estonia; 11 German Center for Infection Research (DZIF), Bonn-Cologne, Germany; 12 Sigmund-Freud-Str. 25, 53127 Bonn, Germany; 13 Meyerhofstraße 1, 69117 Heidelberg, Germany

## Erratum

Following the publication of this article [1], it was brought to our attention that due to miscommunications in the production process, Fig. 1 labels were missing and Fig. 4 labelling was incorrect in the original online version.

The errors:

1. The x and y axis were accidentally omitted from Fig. 1c and d, as well as the key from 1b. The corrected Fig. 1 is presented below:

**Fig. 1:**
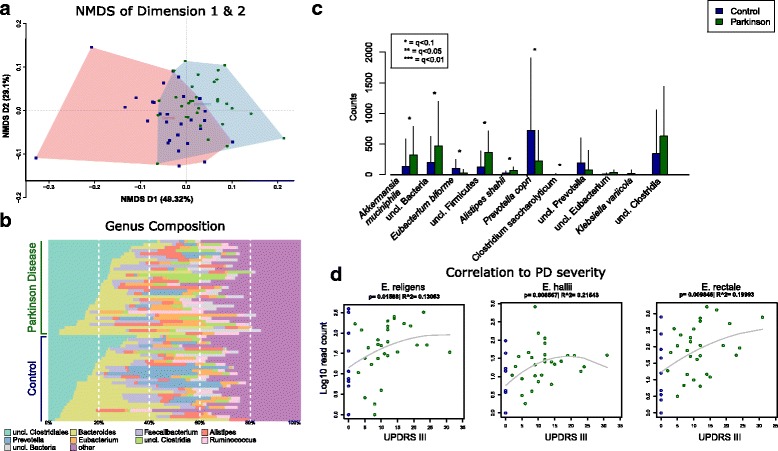
Genus and species level differences in PD participants and controls. **a** NMDS ordination of all samples used in this study, using a Bray–Curtis between-sample distance at genus level. This shows the composition relatedness of samples and that PD samples form a subgroup. Outliers denoted with # took antibiotics in a period of 28–34 days prior to feces sampling. See also Additional file 2 for taxonomic analysis while taking these samples into account. **b** Genus-level sample composition. **c** The most significant species or groups of taxa that could not be further classified. Unclassified Prevotella is not significant after multiple testing, but was implied in PD in several studies (see “Discussion”). **d** Species correlating strongest to PD disease severity (as measured by UPDRS III). Note that after multiple testing correction, these are all q > 0.1
2. MaoB hemmer was erroneously used instead of the English term MaoB inhibitor in Fig. 4. The corrected Fig. 4 is presented below:

**Fig. 4:**
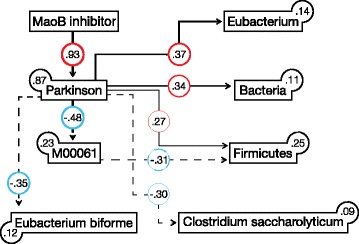
Structural equation modeling (SEM). SEM analysis of PD in relation to key correlating bacterial functions and taxa (MSEA = 0, PCLOSE = 0.79, AIC = 59.385). Values on *paths* and *boxes* are standardized regression and determination coefficients (R2), respectively. *Dashed lines* and *red colors* denote negative relationships. The thickness of lines is proportional to regression coefficients. All relationships are statistically significant (*P* < 0.05, Additional file 5). *AIC* Akaike information criterion, *MSEA* mean square error of approximation, *PCLOSE* probability of close fit

The above errors have been corrected in the original version of this article [1].
