# Supplementary material for: Functional implications of microbial and viral gut metagenome changes in early stage L-DOPA-naïve Parkinson’s disease patients
Source: Genome Med. 2017 Apr 28;9:39. doi: 10.1186/s13073-017-0428-y (PMC5408370; doi:10.1186/s13073-017-0428-y)
Supplement: Supplementary file 1 — Gastrointestinal Symptom Rating Scale (GSRS). Modified version of the GSRS, Gastrointestinal Symptom Rating Scale according to Svedlund et al. 1988, each item was rated from 0 to 3 according to intensity, frequency, duration, or social impact, respectively. (PDF 12 kb) [file 13073_2017_428_MOESM1_ESM.pdf]

Modified version of the Gastrointestinal Symptoms Rating Scale (GSRS) according to *Svedlund et al.* 1988

Item 6: Borborygmus. Representing reports of abdominal rumbling. Rate according to intensity, frequency, duration, and impact on social performance.

0 No or transient borborygmus

1 Occasional troublesome borborygmus of short duration

2 Frequent and prolonged episodes which can be mastered by moving without impairing social performance

3 Continuous borborygmus severely interfering with social performance

Item 7: Abdominal distension. Representing bloating with abdominal gas. Rate according to intensity, frequency, duration and impact on social performance.

0 No or transient distension

1 Occasional discomfort of short duration

2 Frequent and prolonged episodes which can be mastered by adjusting the clothing

3 Continuous discomfort severely interfering with social performance

Item 9: Increased flatus: Representing reports of increased wind. Rate according to intensity, frequency, duration and impact on social performance.

0 No increased flatus

1 Occasional discomfort of short duration

2 Frequent and prolonged episodes interfering with some social performance

3 Frequent episodes seriously interfering with social performance

Item 10: Decreased passage of stools. Representing reported reduced defecation. Rate according to frequency. Distinguish from consistency.

0 Once a day

1 Every third day

2 Every fifth day

3 Every seventh day or less frequently

Item 11: Increased passage of stools. Representing reported increased defecation. Rate according to frequency. Distinguish from consistency.

0 Once a day

1 Three times a day

2 Five times a day

3 Seven times a day or more frequently

Item 12: Loose stools. Representing reported loose stools. Rate according to consistency independent of frequency and feelings of incomplete evacuation.

0 Normal consistency

1 Somewhat loose

2 Runny

3 Watery

Item 13: Hard stools. Representing reported hard stools. Rate according to consistency independent of frequency and feelings of incomplete evacuation.

0 Normal consistency

1 Somewhat hard

2 Hard

3 Hard and fragmented, sometimes in combination with diarrhea

Item 14.: Urgent need for defecation. Representing reports of urgent need for defecation, feelings of incomplete control, and inability to control defecation. Rate according to intensity, frequency, duration and impact on social performance.

0 Normal control

1 Occasional feelings of urgent need for defecation

2 Frequent feelings of urgent need for defecation with sudden need for a toilet interfering with social performance

3 Inability to control defecation

Item 15: Feeling of incomplete evacuation. Representing reports of defecation with straining and a feeling of incomplete evacuation of stools. Rate according to intensity and frequency.

0 Feeling of complete evacuation without straining

1 Defecation somewhat difficult; occasional feelings of incomplete evacuation

2 Defecation definitely difficult; often feelings of incomplete evacuation

3 Defecation extremely difficult; regular feelings of incomplete evacuation
