# Supplementary figures and images for: Functional implications of microbial and viral gut metagenome changes in early stage L-DOPA-naïve Parkinson’s disease patients
Source: Genome Med. 2017 Apr 28;9:39. doi: 10.1186/s13073-017-0428-y (PMC5408370; doi:10.1186/s13073-017-0428-y)

a)

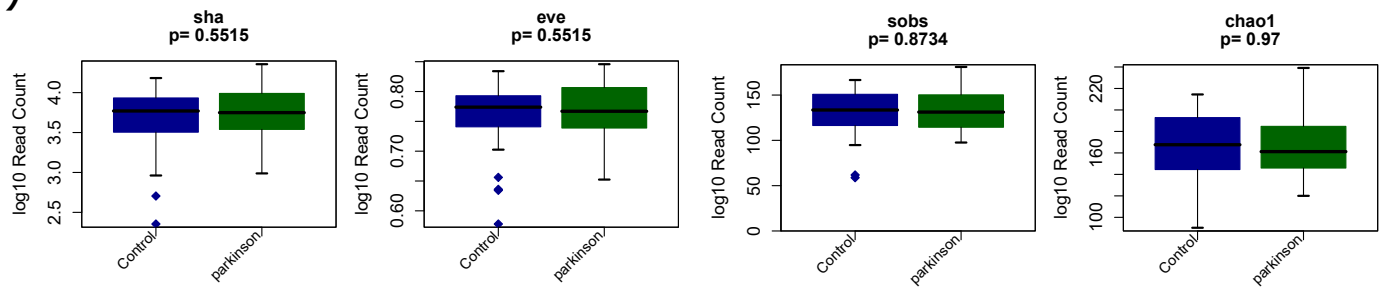

b)

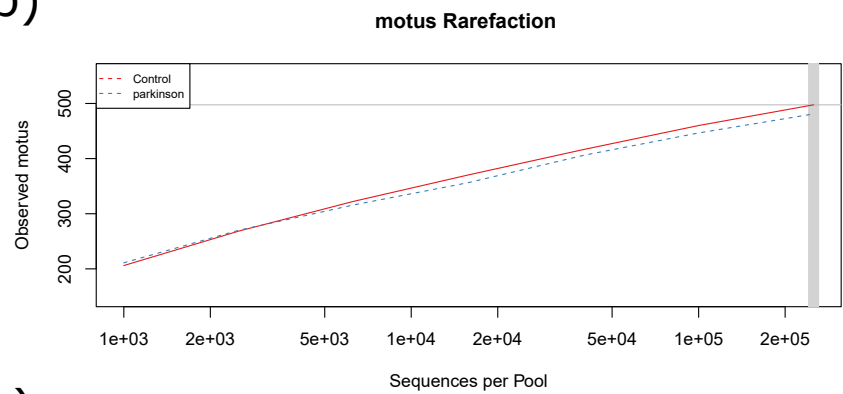

c)

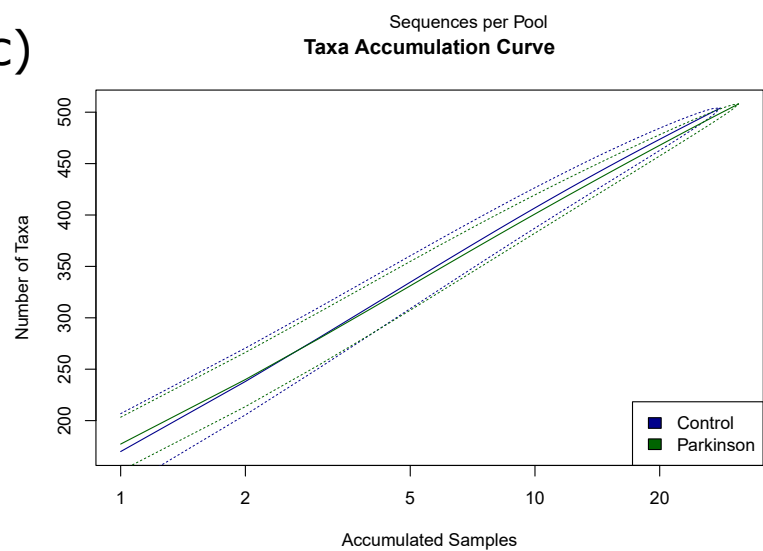

d)

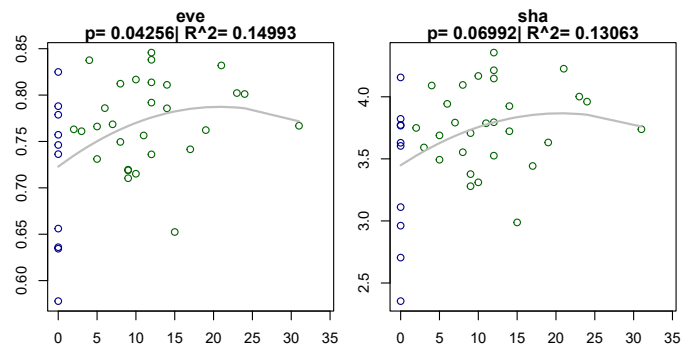

Supplement: Supplementary file 7 — Richness and mOTUs. a Richness of single samples (rarefied to 3000 read coverage) was similar between PD and controls, (b) also, pooling samples and rarefying to different depths showed a similar pattern (rarefied to 251189), as well as (c) measuring the accumulation of new mOTUs when randomly increasing the sampling space. However, evenness and Shannon diversity were positively correlated to UPDRS III. (PDF 64 kb) [file 13073_2017_428_MOESM7_ESM.pdf]

a)

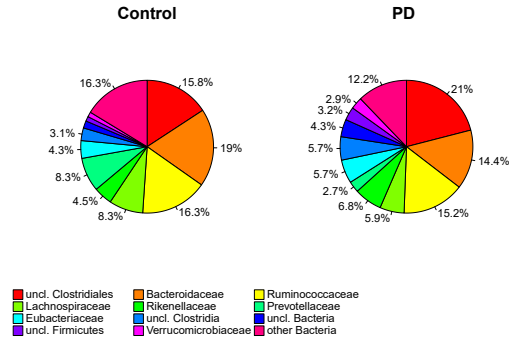

b)

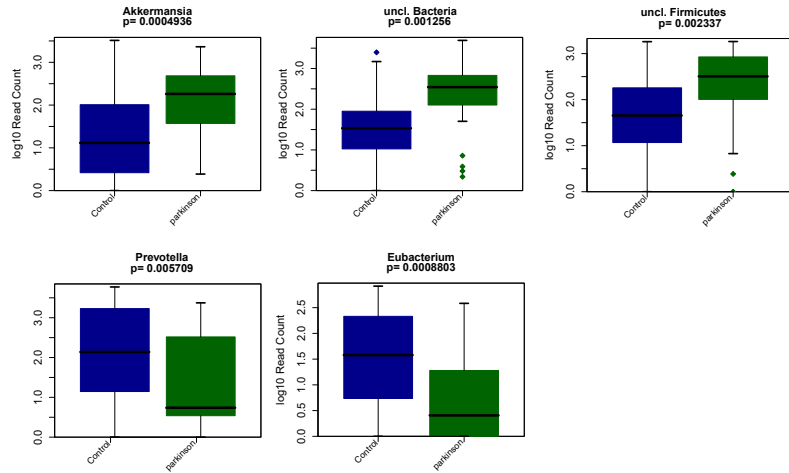

c)

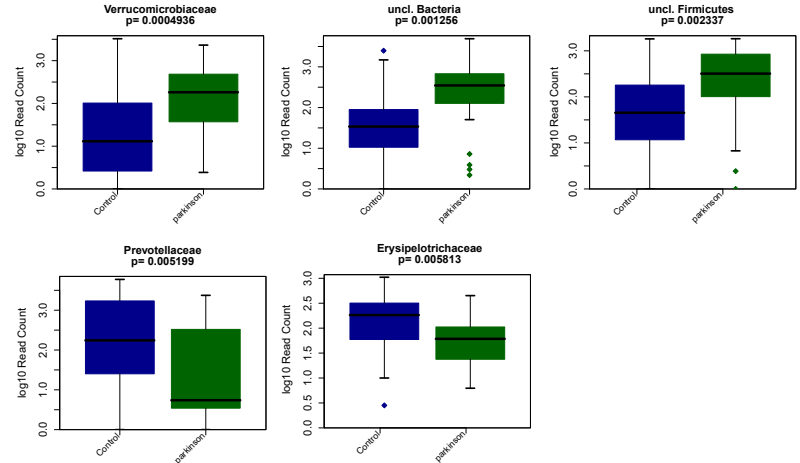

Supplement: Supplementary file 9 — Key taxonomic gut microbiota differences between PD participants and controls. a The 11 most abundant families and their contribution to the gut microbiota displayed in a pie chart. b, c The most significantly different genera and families between PD participants and controls (q < 0.1), confirming previous studies. Note that unclassified bacteria were higher in PD patients. (PDF 88 kb) [file 13073_2017_428_MOESM9_ESM.pdf]

# a) Statins

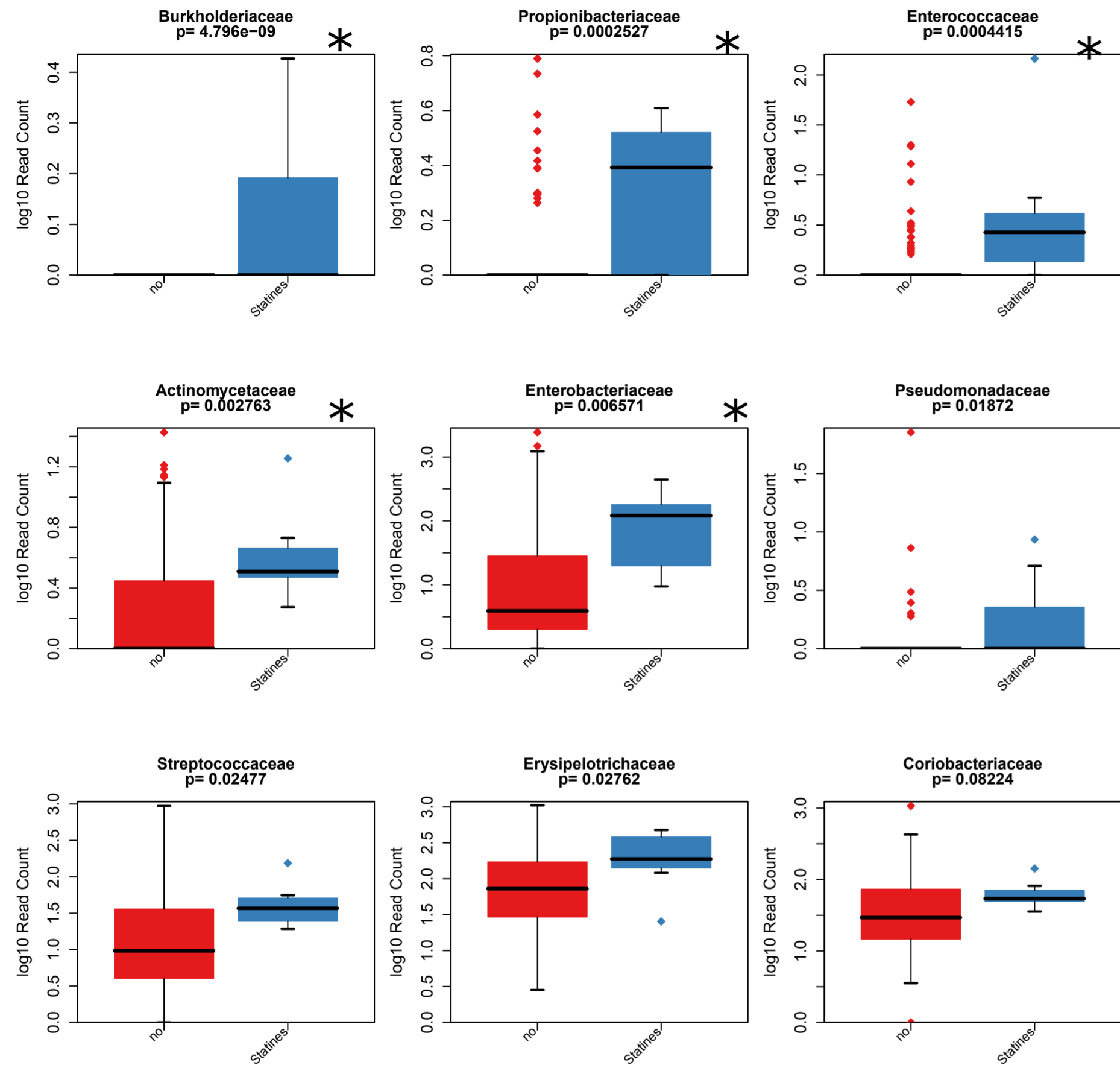

c)

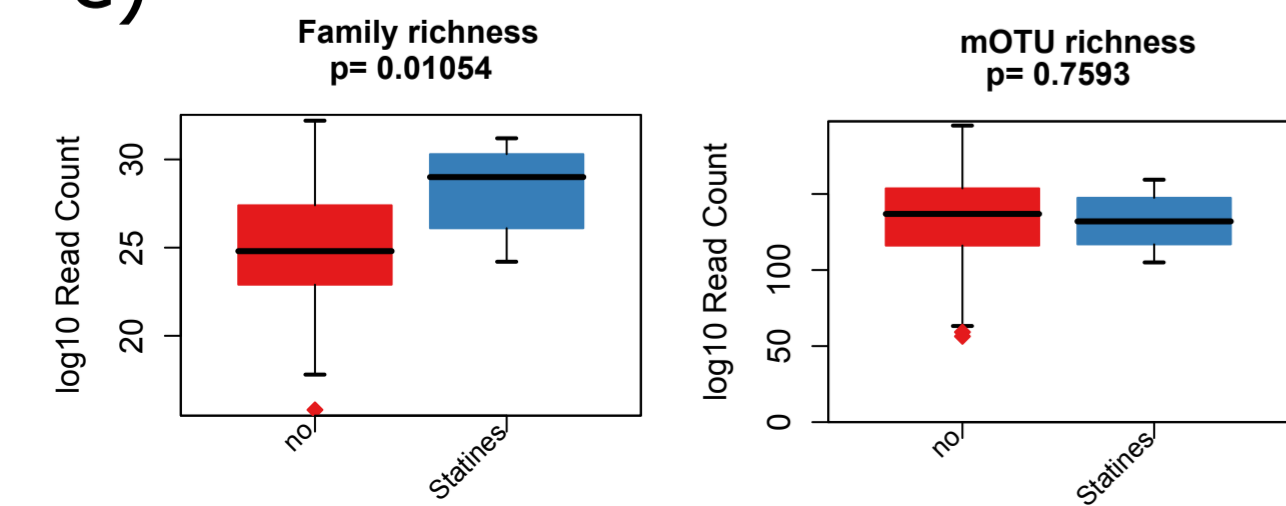

# b) Parkinson medication

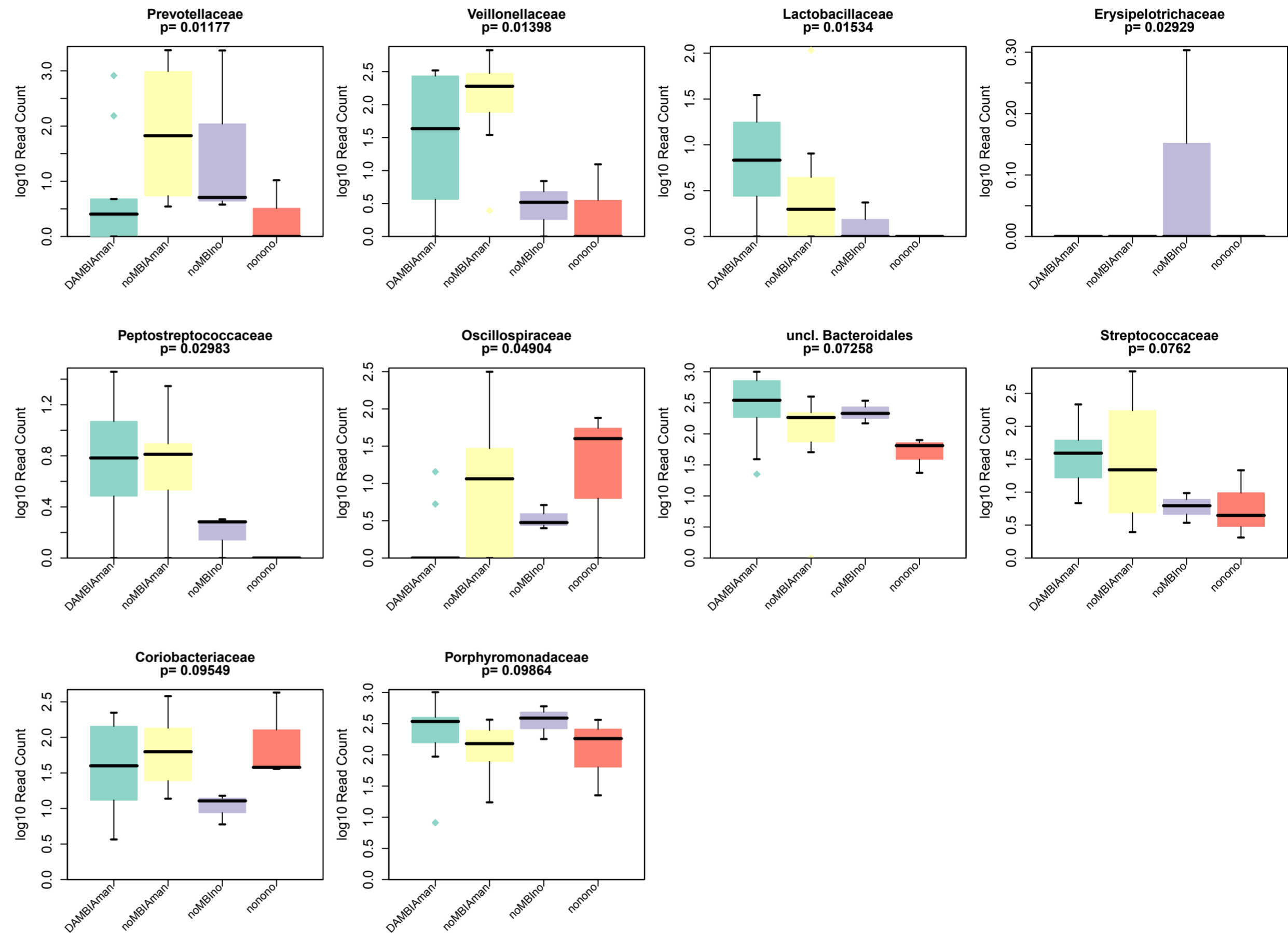

d)

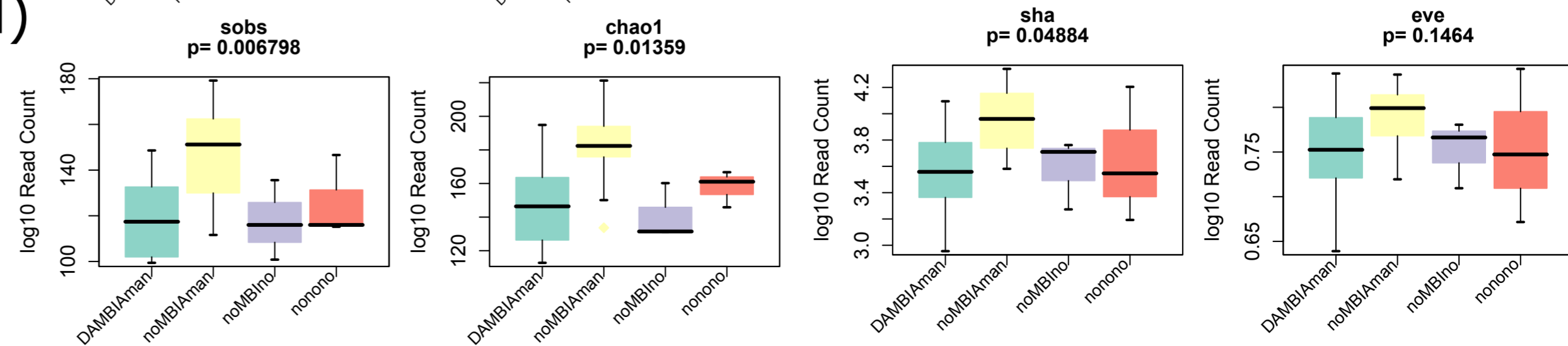

Supplement: Supplementary file 12 — Microbiota differences linked to medication, especially the intake of a statin seemed to have a strong influence on gut microbiota, with (a) five bacterial families as well as (b) family richness significantly different between drug users and medication free patients. c PD medication did not show significant differences in family composition, while (d) gut microbiota mOTU richness differed markedly for patients taking MBI + Aman. DA dopamine agonist, MBI monoamine oxidase inhibitor, Aman amantadine. (PDF 319 kb) [file 13073_2017_428_MOESM12_ESM.pdf]

a)

Viruses  
 $p = 6.677e-05$

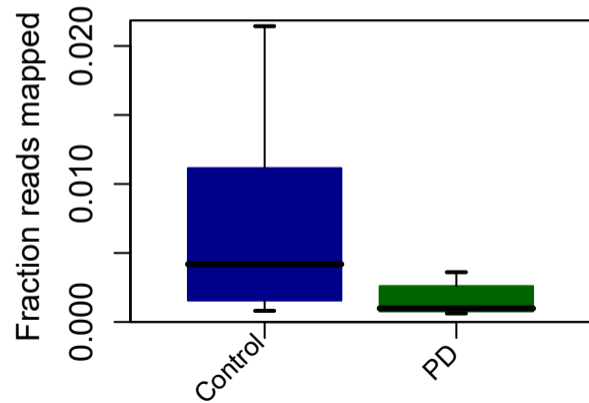

b)

Viruses  
 $p = 6.677e-05$

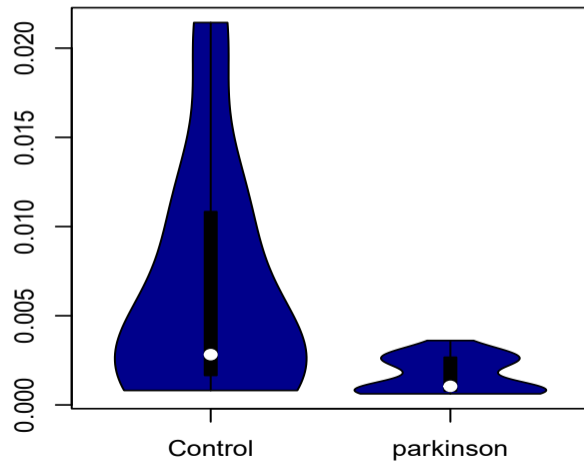

c)

Viruses  
 $p = 0.0009112$

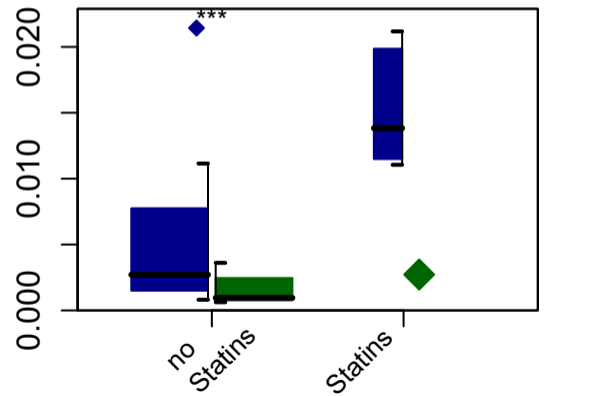

Supplement: Supplementary file 14 — Virus analyses. Fecal virus analyses showed differences between PD and control with (a) PD samples containing fewer amounts of viruses, with the 10th, 25th, 50th, 75th, and 90th quantiles being 0.001, 0.001, 0.001, 0.003, and 0.003 for Parkinson samples and 0.001, 0.002, 0.003, 0.011, and 0.016 for control samples, respectively. b A link to medication with a statin to increase the content of viruses. (PDF 47 kb) [file 13073_2017_428_MOESM14_ESM.pdf]

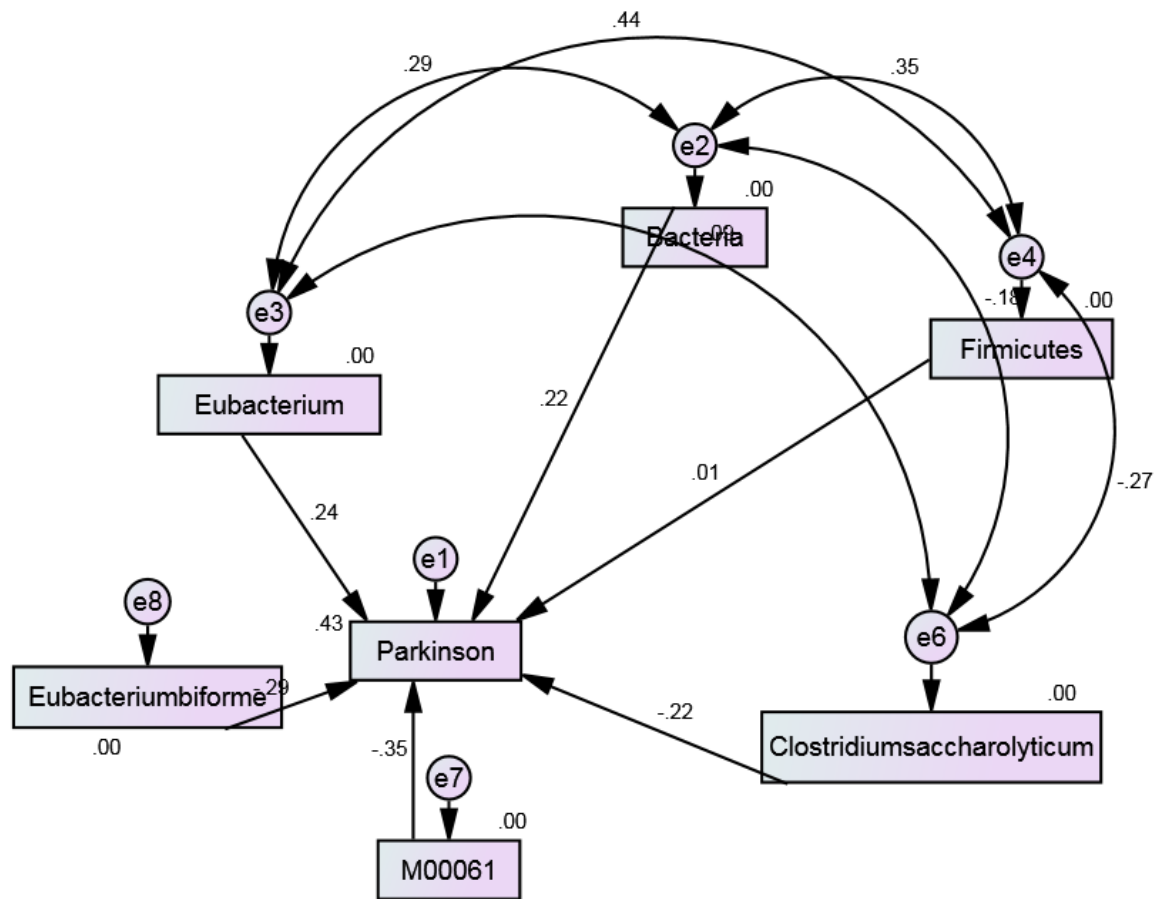

Supplement: Supplementary file 16 — Alternative SEM model. Alternative SEM model of PD in relation to key biotic and abiotic factors (MSEA = 0.113, PCLOSE = 0.138, AIC = 67.447) in which PD is driven by biotic factors, had a worse AIC fit than our proposed SEM modeling of PD disease associations (Fig. 4). AIC Akaike information criterion, MSEA mean square error of approximation, PCLOSE probability of close fit. (PDF 192 kb) [file 13073_2017_428_MOESM16_ESM.pdf]
